# Assessing the Risk of Decrease in Kidney Function in Patients Prescribed Direct-Acting Antivirals for Hepatitis C Utilizing the MID-NET^®^ Medical Information Database Network in Japan

**DOI:** 10.1007/s43441-022-00400-5

**Published:** 2022-04-18

**Authors:** Tomoaki Hasegawa, Sono Sawada, Chieko Ishiguro, Takashi Ando, Kanae Kobayashi, Noriyuki Komiyama, Toyotaka Iguchi, Takahiro Nonaka, Yoshiaki Uyama

**Affiliations:** 1 Office of Medical Informatics and Epidemiology, Pharmaceuticals and Medical Devices Agency, Kasumigaseki 3-3-2, Chiyoda-ku, Tokyo, 100-0013 Japan; 2 Office of Pharmacovigilance II, Pharmaceuticals and Medical Devices Agency, Tokyo, Japan; 3 Present Address: IQVIA Solutions Japan K.K., Tokyo, Japan; 4 Present Address: Section of Clinical Epidemiology, Department of Data Science, Center for Clinical Sciences, National Center for Global Health and Medicine, Tokyo, Japan; 5 Present Address: Department of Health and Medical Innovation, Graduate School of Medicine, Osaka Metropolitan University, Osaka, Japan

**Keywords:** Direct-acting antivirals against hepatitis C, Decreased kidney function, Drug safety, Pharmacovigilance, Pharmacoepidemiology, Real world data

## Abstract

An association between kidney disease and direct-acting antivirals against hepatitis C (DAAs) has been suggested, however the warning on the package insert (PI) of the drug varies among DAAs. In this study, the risk of decreased kidney function associated with DAAs marketed in Japan was investigated to determine whether the risk of kidney disease is a common adverse event and class effect of DAAs. Data for patients who were new users of DAAs marketed in Japan, with eGFR ≥ 45 mL/min/1.73 m^2^ and without specific risk factors, were extracted from the MID-NET^®^ medical information database network in Japan. Changes from the baseline on estimated glomerular filtration rate (eGFR) categories (eGFR ≥ 90, 90 > eGFR ≥ 60, 60 > eGFR ≥ 45, 45 > eGFR ≥ 30, 30 > eGFR ≥ 15, 15 > eGFR; unit: mL/min/1.73 m^2^) were used for evaluating the risk of decreased kidney function. Exposure groups for DAAs and relevant concomitant drugs were categorized into 10 patterns based on the PI. Among the 10 patterns, a significant increase in the incidence rate ratio (*P* < 0.01) was observed in the prescription patterns of concomitant use of telaprevir with peginterferon alpha and ribavirin, concomitant use of daclatasvir hydrochloride with asunaprevir, and ombitasvir hydrate combined with paritaprevir hydrate and ritonavir, which were concomitantly used with ribavirin; such an increase was not observed in the other prescription patterns. The effects of DAAs on kidney function may differ among drugs, suggesting the possibility that the risk of kidney disease is not a class effect of DAAs and should be evaluated individually for each DAA.

## Introduction

Direct acting antivirals against hepatitis C (DAAs) have been widely used for the treatment of hepatitis C in Japan since telaprevir was first approved in 2011 [1]. An association between kidney disease and some DAAs has been suggested, however the warning on the package insert (PI) varies among DAAs [2]. For example, as of March 2018, when this study was planned, telaprevir, simeprevir sodium, and the combination product of ombitasvir hydrate with paritaprevir hydrate and ritonavir included a warning for the risk of kidney disease as “Clinically Significant Adverse Reactions”, while other DAAs such as daclatasvir hydrochloride, vaniprevir, grazoprevir hydrate and elbasvir had no warning in their PIs. On examining the spontaneous adverse reports included in the Japanese Adverse Drug Event Report database (JADER) [3], 399 domestic cases of renal impairment possibly associated with DAAs have been reported between September 2011 (approval of telaprevir as the first DAA in Japan) and August 2018, many of which were in patients prescribed with telaprevir, followed by other products such as the combination products of ombitasvir hydrate with paritaprevir hydrate and ritonavir, ledipasvir acetonate with sofosbuvir, daclatasvir hydrochloride, asunaprevir and sofosbuvir.

In addition to the background described above, since the inhibitory action of telaprevir on renal drug transporters, on organic cation transporter 2 and multidrug and toxin extrusion-type transporter 1 have been postulated to be mechanisms for reducing the estimated glomerular filtration rate (eGFR) [4, 5], other DAAs might also have similar effects on kidney function. Therefore, the PMDA decided to investigate the risk of decreased kidney function associated with DAAs marketed in Japan to consider whether the risk of kidney disease is a common adverse event and class effect of DAAs.

## Methods

### Database

Data from MID-NET^®^, a reliable and valuable database operated by the PMDA with the support of the Ministry of Health, Labour and Welfare of Japan [6, 7], were used for analysis in this study, because it stores electronic medical records, administrative claim data and diagnosis procedure combination data from approximately 5.3 million patients (as of December 2020) in cooperation with 10 healthcare organizations including 23 university hospitals or regional core hospitals. It also has data on eGFR, a relevant indicator for kidney disease, for analysis. The study period was from January 1, 2009 to December 31, 2017, and those data were extracted from MID-NET^®^ in the middle of July 2019.

The utilization of MID-NET^®^ for this study was approved on September 18, 2018, by the expert committee of MID-NET^®^ [8]. Since this study was conducted as an official activity of the PMDA under the Pharmaceuticals and Medical Devices Agency Law (Article 15–5-(c) and (f)) [9], it was not subject to review by institutional review boards [10].

### Cohort

**Fig. 1:**
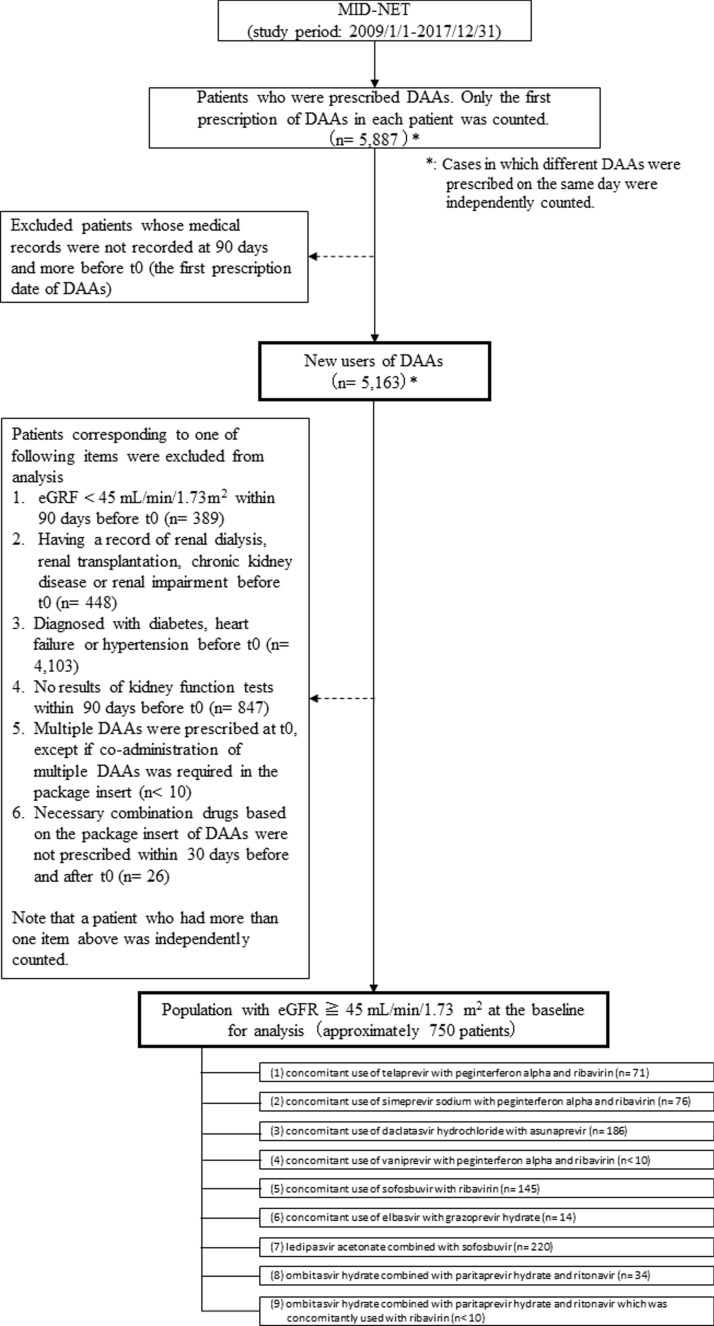
Flowchart for patient selections

The flowchart for patient selection in this study is shown in Fig. 1. First, data for patients who were prescribed DAAs during the study period were extracted from MID-NET^®^. All DAAs marketed in Japan during the study period were investigated in this study, except for the combination product of glecaprevir hydrate with pibrentasvir; this combination product was excluded because an insufficient number of patients for analysis was expected at the planning stage based on the following information: (1) expected patient number of this product was less than 30 in total according to the information available on the website of MID-NET^®^; (2) the end of the study period was only two months after the launch of this combination product. The target DAAs in this study were as follows: telaprevir, simeprevir sodium, asunaprevir, daclatasvir hydrochloride, vaniprevir, sofosbuvir, grazoprevir hydrate, and elbasvir, and the combination products of ledipasvir acetonate with sofosbuvir, ombitasvir hydrate with paritaprevir hydrate and ritonavir, and daclatasvir hydrochloride with asunaprevir and beclabuvir hydrochloride. It should be noted that telaprevir, simeprevir sodium, vaniprevir, the combination product of ombitasvir hydrate with paritaprevir hydrate and ritonavir and the combination product of daclatasvir hydrochloride with asunaprevir and beclabuvir hydrochloride are no longer marketed in Japan [11–15]. Patients whose medical records were not recorded at 90 days or more before *t*_0_ (the first prescription date of DAAs) were excluded to identify new users of each product.

In addition, for analysis, the following exclusion criteria were applied to identify patients with eGFR ≥ 45 mL/min/1.73 m^2^ and without specific risk factors relating to kidney disease: (1) patients with an eGFR within 90 days before *t*_0_ < 45 mL/min/1.73 m^2^; (2) patients with records of renal dialysis, renal transplantation, chronic kidney disease, or renal failure before *t*_0_; (3) patients diagnosed with diabetes, heart failure, or hypertension before *t*_0_; (4) patients who had no eGFR or serum creatinine (sCRE) results within 90 days before *t*_0_; (5) patients who were prescribed multiple DAAs at *t*_0_, except if coadministration of multiple DAAs was required in the PI (i.e., grazoprevir hydrate with elbasvir, daclatasvir hydrochloride with asunaprevir) [16, 17]; and (6) patients who were not prescribed the necessary combination drugs based on the PI of DAAs within 30 days before and after *t*_0_.

### Risk Assessment of Decreased Kidney Function

In the risk assessment of decreased kidney function, we examined changes from the baseline in eGFR categories established by the Kidney Disease Improving Global Outcomes (KDIGO), as shown in Table 1 [18]. The baseline was defined as the eGFR value closest to *t*_0_ during the 90 days before *t*_0_. It should be noted that the exact time of onset of the events in a day was not available in MID-NET^®^. A value of eGFR at *t*_0_ might be observed at either before or after DAA prescription. Therefore, the eGFR value at *t*_0_ was not used for analysis to avoid misinterpretation of results due to a reverse causality on the timing between the onset of decreased kidney function and DAA prescriptions. For analysis, a decrease from the baseline eGFR by one or more eGFR categories (see Table 1) during the follow-up period excluding *t*_0_ were considered as the decreased kidney function. The follow-up period was defined as the period from *t*_0_ to the first of the following dates: occurrence of the outcome, end of the treatment period, date of change to another prescription pattern, or end of the study period (December 31st, 2017). The treatment period consisted of the prescription date (starting date and duration of a prescription) with a 28-day gap and a 7-day grace period for each DAA, except for asunaprevir or grazoprevir hydrate. The treatment period of asunaprevir or grazoprevir hydrate was proportional to that of daclatasvir hydrochloride or elbasvir, because these drugs were only concomitantly used with daclatasvir hydrochloride or elbasvir, respectively. Additional analysis was also conducted by changing the definition to two or more decreases from the baseline in the categories.

**Table 1:** Categories of kidney function based on eGFR

Normal or high	eGFR ≥ 90
Mildly decreased	90 > eGFR ≥ 60
Mildly to moderately decreased	60 > eGFR ≥ 45
Moderately to severely decreased	45 > eGFR ≥ 30
Severely decreased	30 > eGFR ≥ 15
Kidney failure	15 > eGFR

Unit: mL/min/1.73 m^2^

### Definition of Exposure and Control

Based on the PIs of DAAs, exposure groups were categorized into the following 10 patterns: (1) concomitant use of telaprevir with peginterferon alpha and ribavirin (TVR + Peg-IFN + Rib); (2) concomitant use of simeprevir sodium with peginterferon alpha and ribavirin (SMV + Peg-IFN + Rib); (3) concomitant use of daclatasvir hydrochloride with asunaprevir (DCV + ASV); (4) concomitant use of vaniprevir with peginterferon alpha and ribavirin (VAN + Peg-IFN + Rib); (5) concomitant use of sofosbuvir with ribavirin (SOF + Rib); (6) concomitant use of elbasvir with grazoprevir hydrate (EBV + GZR); (7) ledipasvir acetonate combined with sofosbuvir (LDV/SOF); (8) ombitasvir hydrate combined with paritaprevir hydrate and ritonavir (OBV/PTV/r); (9) ombitasvir hydrate combined with paritaprevir hydrate and ritonavir, which was concomitantly used with ribavirin (OBV/PTV/r + Rib); and (10) daclatasvir hydrochloride combined with asunaprevir and beclabuvir hydrochloride (DCV/ASV/BCV). The concomitant drug was decided based on prescription records within 30 days before and after *t*_0_. No patients with the prescription of (10) DCV/ASV/BCV were identified in this study. Thus, further analysis was not conducted on this product. Pattern (7) LDV/SOF was set as the control for analysis, because no safety information relating to kidney disease was included in the serious adverse events section of the PI of this drug, and the greatest number of patients were identified among the patterns without concomitant use of peginterferon alpha or ribavirin, which included a warning for kidney disease in the PI.

### Statistical Analysis

For analysis, median age, sex proportion, and median eGFR at *t*_0_ were calculated for each prescription pattern and were compared among the patterns to understand patients’ background, and the incidence rate ratio of decreased kidney function for each prescription pattern to the control (LDV/SOF) was calculated. When the eGFR value was not available, eGFR value was calculated using the sCRE based on the following formulae which have been widely used in clinical practice in Japan [19, 20]: male, 194 × sCRE^−1.094^ × age^−0.287^ and female, 194 × sCRE^−1.094^ × age^−0.287^ × 0.739. The mean eGFR value was used if multiple eGFR values were recorded on the same day. For calculating patient-years, patient follow-up days were divided by 365.25. The patient-year was calculated as 0 if the follow-up period ended at *t*_0_. SAS version 9.4 (SAS Institute, Cary, NC, USA) was used for all analyses.

## Results

### Cohort

As shown in Fig. 1, after applying all inclusion and exclusion criteria, approximately 750 patients were included in the analysis. Although 5163 patients were identified as new users of DAAs, many patients were excluded because of the presence of concomitant diseases, such as diabetes, heart failure, or hypertension (*n* = 4103), or a lack of eGFR and sCRE results (*n* = 847). It should be noted that since a patient who met more than one exclusion criterion was independently counted for each item, these numbers include duplicate counts of a patient.

### Risk Comparison of Decreased Kidney Function Among DAAs

Baseline patient characteristics are shown in Table 2. The analyzed population generally included more female and elderly patients, but there were no significant differences in eGFR (*P* > 0.05, Wilcoxon rank sum test, vs. the control) or sex ratio (*P* > 0.05, Fisher’s exact test, vs. the control) among the prescription patterns, although slight differences were observed in age. In the population for analysis, the median (interquartile range) of the follow-up period was 87 days (34–90 days) and the median number (interquartile range) of kidney function tests per patient was 4 (2–6) during the follow-up period.

**Table 2:** Baseline patient characteristics in the primary analysis (risk comparison of decreased kidney function among DAAs in patients with eGFR ≥ 45 mL/min/1.73 m^2^)

Prescription pattern^a^	Median age, years (interquartile range)	Female, *n* (%)	Median eGFR, mL/min/1.73 m^2^ (interquartile range)
(1) TVR + Peg-IFN + Rib (*n* = 71)	60 (53–64)	36 (50.7%)	74.8 (66.2–80.0)
(2) SMV + Peg-IFN + Rib (*n* = 76)	62 (56–69)	42 (55.3%)	76.1 (64.6–85.0)
(3) DCV + ASV (*n* = 186)	71 (64–77)	106 (57.0%)	73.8 (63.3–83.5)
(4) VAN + Peg-IFN + Rib^b^ (*n* < 10)	63 (59–67)	< 10 (100.0%)	64.0 (54.0–74.0)
(5) SOF + Rib (*n* = 145)	60 (50–72)	86 (59.3%)	73.5 (64.3–87.0)
(6) EBV + GZR^b^ (*n* = 14)	65 (49–80)	< 10 (NA)	74.5 (70.0–80.4)
(7) LDV/SOF (*n* = 220)	66 (56–76)	127 (57.7%)	73.0 (63.7–83.5)
(8) OBV/PTV/r (*n* = 34)	69 (57–74)	20 (58.8%)	73.9 (64.6–90.4)
(9) OBV/PTV/r + Rib^b^ (*n* < 10)	48 (42–53)	< 10 (NA)	92.1 (91.9–92.3)

*NA* not available, *TVR* telaprevir, *Peg-IFN* peginterferon alpha, *Rib* ribavirin, *SMV* simeprevir sodium, *DCV* daclatasvir hydrochloride, *ASV* asunaprevir, *VAN* vaniprevir, *SOF* sofosbuvir, *EBV* elbasvir, *GZR* grazoprevir hydrate, *LDV* ledipasvir acetonate, *OBV* ombitasvir hydrate, *PTV* paritaprevir hydrate, *r* ritonavir

^a^“+” represents concomitant use of a drug and “/” represents a combination product

^b^It was shown as an aggregated value based on the MID-NET^®^ publication rule

**Fig. 2:**
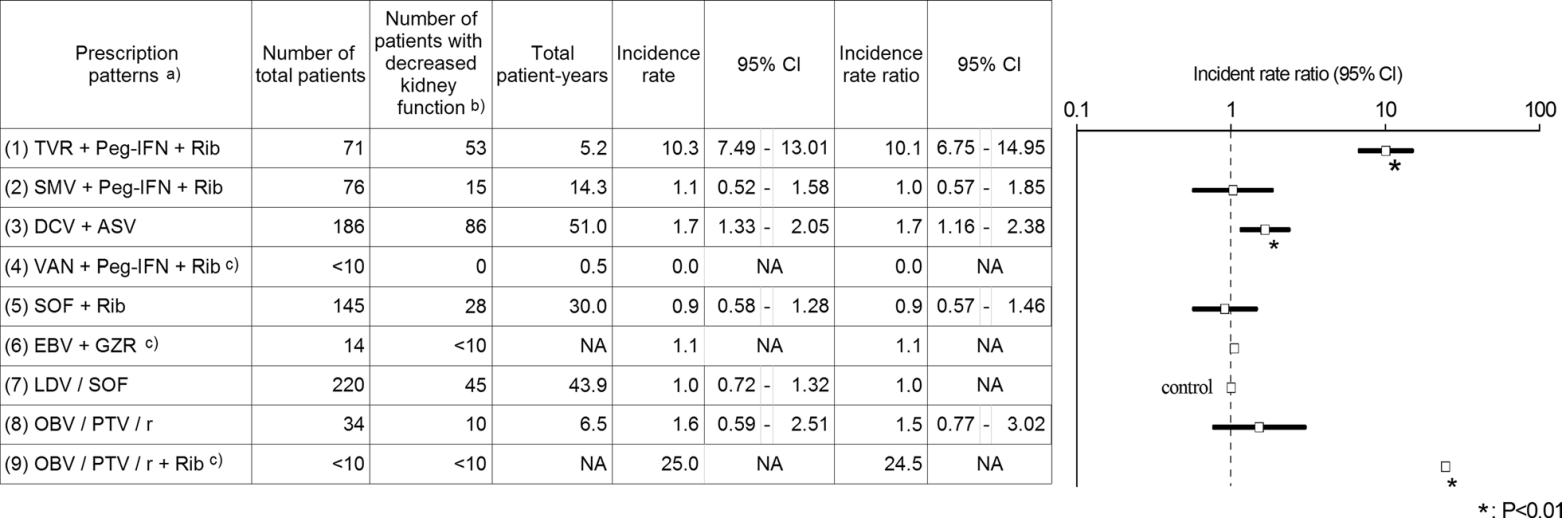
Incidence rate of decreased kidney function for each prescription pattern and its ratio to the control. ^a^“+” represents a concomitant use of a drug and “/” represents combination product. *NA* not available. *TVR* telaprevir, *Peg-IFN* peginterferon alpha, *Rib* ribavirin, *SMV* simeprevir sodium, *DCV* daclatasvir hydrochloride, *ASV* asunaprevir, *VAN* vaniprevir, *SOF* sofosbuvir, *EBV* elbasvir, *GZR* grazoprevir hydrate, *LDV* ledipasvir acetonate, *OBV* ombitasvir hydrate, *PTV* paritaprevir hydrate, *r* ritonavir. ^b^Defined as a decrease from the baseline eGFR by one or more eGFR categories (see Methods and Table 1). ^c^It was shown as an aggregated value based on the MID-NET^®^ publication rule

The incidence rate ratio of decreased kidney function, defined as one or more decreases from the baseline in eGFR categories (see methods), of each prescription pattern to the control (LDV/SOF) is shown in Fig. 2. A significant increase in the incidence rate ratio was observed in the prescription pattern of (1) TVR + Peg-IFN + Rib, (3) DCV + ASV, and (9) OBV/PTV/r + Rib (*P* < 0.01), whereas such an increase was not observed in the other prescription patterns.

When the definition of decreased kidney function was changed to two or more decreases from the baseline in eGFR categories, a significantly increased risk was also observed for (1) TVR + Peg-IFN + Rib (incidence rate ratio: 41.75, 95% CI: 9.84–177.09). This increased risk was not clear for the other prescription patterns because only a small number or no observed cases of decreased kidney function were identified, resulting from a lower frequency of the event (data not shown).

## Discussion

The present study was conducted to evaluate the risk of decreased kidney function in patients prescribed DAAs marketed in Japan. A significantly increased risk in comparison with the control ((7) LDV/SOF) was observed for (1) TVR + Peg-IFN + Rib, (3) DCV + ASV, and (9) OBV/PTV/r + Rib. These results support previous findings regarding the risk of kidney disease associated with TVR, DCV + ASV, and OBV/PTV/r, such as a decline of eGFR and an occurrence of acute kidney injury during DAA treatment [21, 22]. The PI of these DAAs already include a warning on kidney disease [2]. In the case of (1) TVR + Peg-IFN + Rib and (9) OBV/PTV/r + Rib, the observed risk may be also associated with Peg-IFN or Rib, whose risk of kidney disease is described in the respective PIs. In contrast, no increased risk of decreased kidney function was observed for other prescription patterns, such as (2) SMV + Peg-IFN + Rib, (4) VAN + Peg-IFN + Rib, (5) SOF + Rib, (6) EBV + GZR, and (8) OBV/PTV/r. This may suggest that the risk of decreased kidney function varies among DAAs and is not a class effect of DAAs, although the risk is undeniable because the control itself (the prescription pattern of (7) LDF/SOF) may cause decreased kidney function. In fact, the PI for simeprevir and (8) OBV/PTV/r includes a warning on the risk of kidney disease. Thus, further investigations are necessary to clarify the degree of risk. In addition, the lack of increased risk, even in the case of the prescription patterns with Peg-IFN or Rib may imply that Peg-IFN or Rib is not the key factor for the risk identified in this study. It should be noted that the results for (4) VAN + Peg-IFN + Rib, (6) EBV + GZR, and (9) OBV/PTV/r + Rib may not be stable due to the smaller sample size (< 20) and should be interpreted cautiously.

The strength of this study was its utilization of eGFR as an objective variable to examine decreased kidney function based on data from MID-NET^®^, which has been reported to be a reliable database [6]. As a limitation, patient background was not adjusted in detail when comparing the risk of DAAs, although no major differences in terms of baseline eGFR were confirmed. In addition, this study only targeted patients with eGFR ≥ 45 mL/min/1.73 m^2^ at baseline and excluded patients who had risk factors (diabetes, heart failure or hypertension) related to kidney disease. Thus, we believe that no further adjustments are necessary to interpret the results of this study. Another limitation was that some of the patients who were prescribed DAAs were excluded due to a lack of results on kidney function tests within 90 days before *t*_0_. This may affect the results in a part, although such effects could be negligible or limited because the number of such patients was limited and patients with a higher risk of kidney disease would be carefully monitored in clinical practice, including monitoring of eGFR. Another study may also be necessary to examine a reversibility of decreased kidney function by DAAs, which was out of the scope in this study.

The PMDA conducted a safety assessment regarding kidney disease associated with DAAs based on the results of this study, and concluded that no new signals were identified and that the study results support the current safety warnings in the PIs so that no additional safety measures are required at present. However, as discussed above, these different effects of DAAs on kidney function should be recognized by marketing authorization holders of DAAs and health care professionals for its proper use, although more research will be necessary for examining specific measures in clinical practice.

## Conclusion

The effects of DAAs on kidney function may differ among drugs, suggesting the possibility that the risk of kidney disease is not a class effect of DAAs and should be evaluated individually for each DAA.
